# Entropy Profiling: A Reduced—Parametric Measure of Kolmogorov—Sinai Entropy from Short-Term HRV Signal

**DOI:** 10.3390/e22121396

**Published:** 2020-12-10

**Authors:** Chandan Karmakar, Radhagayathri Udhayakumar, Marimuthu Palaniswami

**Affiliations:** 1 School of Information Technology, Deakin University, Geelong VIC 3216, Australia; radhagayathri.udhayakumar@deakin.edu.au; 2 Department of Electrical & Electronic Engineering, The University of Melbourne, Parkville VIC 3010, Australia; palani@unimelb.edu.au

**Keywords:** entropy profiling, heart rate variability, short-term HRV time series, irregularity analysis, complexity analysis, tolerance, non-parametric K-S entropy

## Abstract

Entropy profiling is a recently introduced approach that reduces parametric dependence in traditional Kolmogorov-Sinai (KS) entropy measurement algorithms. The choice of the threshold parameter *r* of vector distances in traditional entropy computations is crucial in deciding the accuracy of signal irregularity information retrieved by these methods. In addition to making parametric choices completely data-driven, entropy profiling generates a complete profile of entropy information as against a single entropy estimate (seen in traditional algorithms). The benefits of using “profiling” instead of “estimation” are: (a) precursory methods such as approximate and sample entropy that have had the limitation of handling short-term signals (less than 1000 samples) are now made capable of the same; (b) the entropy measure can capture complexity information from short and long-term signals without multi-scaling; and (c) this new approach facilitates enhanced information retrieval from short-term HRV signals. The novel concept of entropy profiling has greatly equipped traditional algorithms to overcome existing limitations and broaden applicability in the field of short-term signal analysis. In this work, we present a review of KS-entropy methods and their limitations in the context of short-term heart rate variability analysis and elucidate the benefits of using entropy profiling as an alternative for the same.

## 1. Introduction

Heart rate variability (HRV), a variation in the time interval between successive heart beats, is a prognostic indicator of various physiological conditions, such as disease, stress, aging, fitness and gender [1,2,3]. A heart rate signal is non-stationary [4], and its variability follows non-linear dynamics that are both complex and chaotic in nature [5,6]. HRV analysis is a popular non-invasive technique to assess cardiac health and other significant physiological conditions. Every physiological condition is linked to a change in heart rate variability and its complexity, thereby making HRV analysis a significant clinical tool. The physiological process of HRV being highly non-linear in nature cannot be contained fully by linear approaches. This necessitates the use of non-linear dynamic methods to carry out HRV analysis. Of these, the commonly used statistical measures are correlation dimension, Lyapunov exponents, entropy algorithms and Poincare plot.

Due to procedural and economical constraints, it is generally preferred to conduct HRV analysis on “short-term” data. The use of short-term data facilitates quick signal acquisition, and hence supports a patient-friendly procedure, and reduces diagnosis and consultation times, leading to reduced medical expenses; and also will have minimal influences from motion artifacts [7]. To analyze short-term HRV data, the most popularly used non-linear tools are the KS-entropy (Kolmogorov-Sinai) methods. Non-linear KS-entropy algorithms, such as approximate entropy (*ApEn*) and sample entropy (*SampEn*), are efficient in dealing with short-term data, but their dependence on input parameters affects the quality of information retrieval to a great extent. Elimination of the parametric dependence of such methods will prove beneficial in the context of information retrieval from short-term heart rate data.

The issue of parametric dependence has been dealt in different ways. Entropy methods have been modified to (a) reduce the impact of parameters on them [8], (b) formulate a process for logical selection of parameters rather than a random selection [9], eliminate parameters from their formulations [10] and so on. However, the actual source of the issue remains unexplored. A single choice of parameter gives a single entropy estimate that does not always represent complete information present in the HRV. Using multiple parameter choices would mean calculating multiple entropy estimates (entropy at all potential parameter values). This generates a profile of entropy values, in contrast to a single estimate of entropy. This certainly means enhanced information retrieval from the signal.

**Figure 1:**
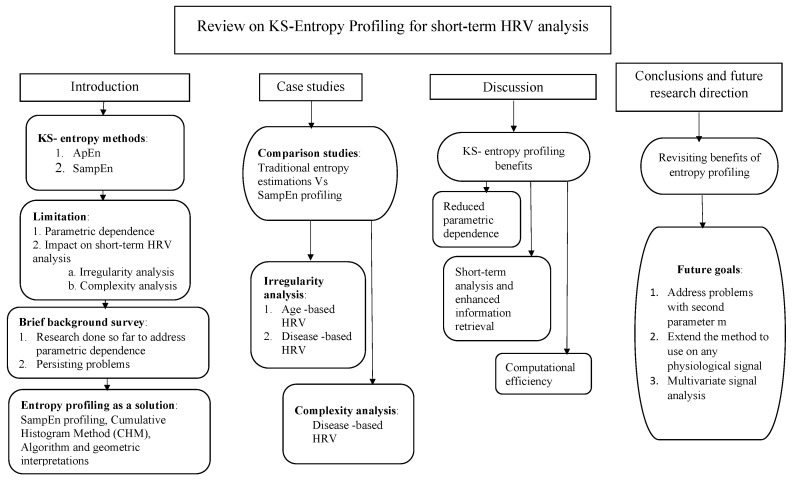
Flowchart of contents in this review paper. The content has been organized under four major branches: (1) an introduction and background; (2) case studies and results; (3) a detailed discussion of the results and impacts; (4) conclusive comments and future research directions.

How entropy profiling can be done efficiently and how the profiling can be used to improve short-length HRV signal analysis have been the main contributions of our research. To the best of our knowledge, there has not been done any extensive review on the means to address entropy methods’ parametric dependence. Having introduced a novel solution to address the same, we find the time and opportunity suitable to present a detailed review on KS-entropy methods, their limitations, research progress and our novel idea of entropy profiling. A flow chart of contents in this review paper is given in Figure 1. The content could be seen as divided into four main branches: (1) an introduction and necessary background information; (2) case studies and results; (3) a detailed discussion of the results and impacts; (4) conclusive comments and future research directions.

This paper presents a review on the newly introduced method of “entropy profiling” meant for the analysis of short-term HRV signals. Entropy profiling was developed as an alternative or improvement to existing KS-entropy methods. Contributions of this review can be summarized as:

- An explanation of the parametric dependency of KS-entropy measures and its impact on short-term HRV analysis.
- A summary of approaches proposed in the last two decades to address parametric dependency and a summary of persisting issues.
- Explaining the concept, formulation and geometric meaning of entropy profiling as a solution to persisting issues in the context of KS entropy for short-term HRV analysis.
- Summary of significant research directions based on the provided entropy profiling solution.

## 2. Kolmogorov Entropy Methods

Kolmogorov–Sinai (KS) entropy was formulated by [11] to quantify signal/time-series regularity. This was further improved by [11,12,13,14] and has since then been quite popular.

The KS entropy can be defined as the conditional probability of two segments of a time-series (of data length *N*) matching at a length m+1 if they match at a length *m*, where the matching of segments is decided by a threshold *r*, given by r=ktimesSDofsignal, where *k* can take values between 0 and 1. The KS-entropy measure is evaluated for the parameter limits r→0, m→∞ and N→∞ [13,14]. The KS entropy of a given time series {x(n);1≤n≤N} can be formulated as shown below.

For a given value of the embedding dimension *m*:

1. Form (N−m+1) vectors of length *m* each, given by
  $$\left\{X_{i}^{m}:1\leq i\leq \left(N-m+1\right)\right\}$$
  (1) $$\mathrm{where},\;X_{i}^{m}=\left\{x(i+k):0\leq k\leq m-1\right\}.$$
2. Similarly, form (N−m) vectors of length (m+1) each, given by
  $$\left\{X_{i}^{m+1}:1\leq i\leq \left(N-m\right)\right\}$$
  (2) $$\mathrm{where},\;X_{i}^{m+1}=\left\{x(i+k):0\leq k\leq m\right\}.$$
3. Take each $X_{i}^{m}$ vector of step 1 as a template vector and find its distance from every vector of $X_{j}^{m}$, where the distance is given by
  (3) $$\begin{array}{cc} d_{ij}^{m} & =\{\max|X_{i}^{m}-X_{j}^{m}\left|:\;1\leq j\leq (N-m+1)\right\}. \end{array}$$
4. Let $B_{i}$ be the count of the number of vectors of $X^{m}$ which lie within a distance *r* of the vector $X_{i}^{m}$.
5. The probability of a vector $X_{j}^{m}$ to lie within a distance *r* of the vector $X_{i}^{m}$ is given by
  (4) $$\begin{array}{cc} C_{i}^{m}\left(r\right) & =\frac{B_{i}}{N-m+1}. \end{array}$$
6. Finally, we take the average of the natural logarithms of these probabilities for i≤(N−m+1) as
  (5) $$\begin{array}{cc} \Phi^{m}\left(r\right) & =\frac{1}{N-m+1}\left[\begin{array}{c} N-m+1\\ \sum \\ i=1 \end{array}\ln C_{i}^{m}\left(r\right)\right]. \end{array}$$
7. Repeating steps 3 to 6 for the (m+1) dimension, we get
  (6) $$\begin{array}{cc} \Phi^{m+1}\left(r\right) & =\frac{1}{N-m}\left[\begin{array}{c} N-m\\ \sum \\ i=1 \end{array}\ln C_{i}^{m+1}\left(r\right)\right]. \end{array}$$

The above steps are repeated for limiting values of N,mandr, till a convergence of $\left[\Phi^{m}\left(r\right)-\Phi^{m+1}\left(r\right)\right]$ is obtained and this converged value corresponds to KS entropy or simply Entropy of the signal.

(7) $$\begin{array}{c} Entropy\left(N,m,r\right)=\begin{array}{c} \lim\\ r\to 0 \end{array}\begin{array}{c} \lim\\ m\to \infty \end{array}\begin{array}{c} \lim\\ N\to \infty \end{array}\left[\Phi^{m}\left(r\right)-\Phi^{m+1}\left(r\right)\right]. \end{array}$$

The existence of such strange attractors makes the method computationally expensive in most cases.

### 2.1. Approximate Entropy (ApEn)

In 1991, S.M. Pincus [13] introduced a new regularity statistic called “approximate entropy” which is an approximated version of Kolmogorov-Sinai (KS) [13,14] entropy. ApEn is the KS entropy estimated at a single value of *r*, *m* and *N*.

Given a time series {x(n);1≤n≤N} of length *N*,

(8) $$\begin{array}{cc} ApEn(m,r) & =\Phi^{m}\left(r\right)-\Phi^{m+1}\left(r\right), \end{array}$$

where $\Phi^{m}\left(r\right)$ and $\Phi^{m+1}\left(r\right)$ are calculated as per steps 1 to 7 of the KS-entropy method. Single values of embedding dimension *m* and tolerance *r* are chosen from recommended ranges of {2,3,4} and {[0.1–0.2] times SD of signal} respectively.

### 2.2. Sample Entropy (SampEn)

ApEn was considered a biased measure since its computation included self-matches of sub-sequences i.e., while calculating vector distances from Equation (3), distance of every vector from itself is also included in the set. These self matches do not contribute logically to the procedure. They are included only to prevent entropy values from being undefined ($min(B_{i})=1$ in Equation (4). However, as a consequence, ApEn always showed higher regularity than the signal contained. Yet another issue was ApEn being highly influenced by data length *N* [15,16,17]. In order to rectify these issues, Richman and Moormann, in 2000, proposed “sample entropy” [16]. Sample entropy completely eliminated self matches from the evaluation process and was observed to be more consistent in comparison to ApEn [15,17]. However, this increase in accuracy and consistency was at the cost of getting undefined values of SampEn at lower values of *N* and *r* and higher values of *m* [10,18].

SampEn formulation is a slightly modified version of ApEn formulation. Here, self-matches in vectors are eliminated from the formulation and also the same number of template vectors are generated in both *m* and m+1 dimensions. For a given time series data of length *N*, sample entropy is calculated as

(9) $$\begin{array}{cc} SampEn & =\ln\frac{\Phi^{m}\left(r\right)}{\Phi^{m+1}\left(r\right)} \end{array}$$

where

(10) $$\begin{array}{cc} \Phi^{m}\left(r\right) & =\frac{1}{N-m}\begin{array}{c} N-m\\ \sum \\ i=1 \end{array}C_{i}^{m}\left(r\right) \end{array}$$

(11) $$\begin{array}{cc} \Phi^{m+1}\left(r\right) & =\frac{1}{N-m}\begin{array}{c} N-m\\ \sum \\ i=1 \end{array}C_{i}^{m+1}\left(r\right) \end{array}$$

$C_{i}^{m}\left(r\right)$ being the probability of a vector $X_{j}^{m}$ to lie within a distance *r* of the vector $X_{i}^{m}$, 1≤j≤(N−m), j≠i. Similarly, $C_{i}^{m+1}\left(r\right)$ is the probability of a vector $X_{j}^{m+1}$ to lie within a distance *r* of the vector $X_{i}^{m+1}$, 1≤j≤(N−m), j≠i.

Several other modified and improved versions of KS entropy such as multiscale entropy [19,20], cross entropy [13,21], permutation-based KS entropy [22] and so on have been introduced and tried on real-time biomedical data sets.

## 3. Limitations of KS-Entropy Methods

KS-entropy methods have had several limitations right from their inception. The first and foremost being the bias in ApEn due to the calculation of self-matches [23]. As a result, SampEn was introduced and widely accepted as a better alternative. What still remains as the most crucial part of calculating these entropy measures (ApEn and SampEn) is the selection of appropriate parameter values for *m* and *r* [9,24,25,26,27]. If not chosen with a certain level of discretion, these parameters may give completely wrong estimates of signal regularity [28]. For most signals, the recommended choice of *m* is 2 and *r* is 0.1–0.25 times the standard deviation (SD) of the signal. However, the choice has been always inconsistent over different data groups. Many studies have reported the generation of misleading and ambiguous results when using prescribed parameter values. Additionally, different values of *m* and *r* lead to different entropy values for the same time-series data [14,16]. This implies that the selection of [m,r] in itself introduces a bias in the entropy calculation. Researchers have attempted to either eliminate or reduce the impact of parameters [m,r] on entropy procedures.

### 3.1. Tolerance *r*

The *r* parameter being the most critical of all happens to be the major source of inconsistencies reported so far. When using medical signals, Pincus et al. suggested a value of *r* in the range of 0.1 to 0.25 times standard deviation (*SD*) of the time series signal [14]. However, when choosing *r* in this prescribed range, several experimental failures were reported [9,24,25,26,27]. Researchers realized that the suggested *r* range could not be generalized across different data. Thus, some tried to come up with new techniques to define a more logical selection of *r*. Others tried to lessen the role of *r* when implementing entropy formulations.

Lu et al. [9] came up with the idea of $r_{max}$, the *r* value yielding maximum entropy. $r_{max}$ was believed to be a more rational choice of *r* when estimating approximate entropy of a signal. However, in addition to the fact that the idea could not be implemented on other statistics such as SampEn [29], the formulation to calculate $r_{max}$ was inconsistent across data groups [29]. Chen et al. [8] proposed fuzzy entropy (FuzzyEn). The method made use of a similarity function based on fuzzy membership, to asses sub-sequence matching of vectors. In the case of ApEn and SampEn, a Heaviside function performed the action instead. Overall, the impact of *r* was much less on FuzzyEn, compared to that on precursors such as ApEn and SampEn. Additionally, unlike SampEn, FuzzyEn did not produce indefinite values of entropy at any point of *r* [8,18].

Despite these new solutions, the ground problem remains. Entropy values, be it ApEn, SampEn or FuzzyEn, tend to differ with a change in the *r* choice, for the same given signal [10,30]. Thus, arriving at the most right choice of the parameter continues to be critical. Lessening the influence of *r* on formulations alone does not address the main problem.

In 2015, another group of researchers tried to fully eliminate the *r* parameter from their new entropy formulation called distribution entropy (DistEn) [10]. In place of *r*, they used a new parameter *M*, denoting the number of bins in the empirical probability distributions used to compute DistEn. *M* is not as critical a parameter as *r*, therefore the method has definitely provided a significant direction to the existing problem. However, again DistEn cannot be considered the right solution in this case because it is a Shannon-entropy based formulation, whereas ApEn, SampEn and FuzzyEn are KS-entropy based. These two families of entropy estimation that have formative differences cannot be compared in the same scale. An elaborate discussion on all the above methods is given in upcoming sections of the manuscript.

### 3.2. Impact of *r* Choice in Short-Term HRV Analysis

Since their inception, ApEn and SampEn have extensively found application in extracting regularity based information from short-term (2–15 minutes of data) HRV signals. A few of the applications include detection of cardiac arrhythmias such as atrial fibrillation [31,32] and cardiac dysfunctions such as congestive heart failure [33,34] and myocardial infarction [35,36], age based and gender based discrimination of HRV signals [7,37,38,39], fetal heart rate analysis [36,40], sleep apnea detection [15] and so on. Despite being widely used for short-term HRV analysis, existing questions and problems associated with the methods are as follows:

1. ApEn and SampEn are known to produce misleading regularity results owing to their parametric restrictions, particularly the *r* parameter. A wrong choice of *r* may give completely unreal regularity estimates for a signal. Thus, the current scheme of *r* selection in entropy methods makes regularity estimation unreliable.
2. A lack of logical flexibility in choosing *r* places its share of restrictions on signal length as well. For the currently available mode of *r* selection, at least 1000 data points (≃15 min data) from the incoming signal are required for analysis. Thus, is ApEn or SampEn incapable of handling signals below 1000 data points? This cannot be explored or concluded unless the lameness in *r* selection procedure is addressed. Thus, how do we make entropy methods suitable for use on short-length (<15 min) data?

Reportedly, an *r* choice from 0.1–0.25 times SD (standard deviation) of the signal is recommended for signals of length between 50 to 5000, while estimating ApEn at an m=1 or 2 [23]. This was followed in SampEn estimation too. However, various studies started reporting the inappropriateness of using such a generalized *r* choice across data sets and urged the need for proper discretion while making the choice [9,28,30,40,41].

### 3.3. HRV Complexity Analysis

ApEn and SampEn are primarily “irregularity” statistics. They assess a signal’s state of orderliness (or chaos) by surveying existential patterns interpreted from the signal. An “irregular” signal may not always be associated with a high level of “complexity” and vice-verse. For example, when an original time series (say, one that represents an underlying complex system) is randomized to form its surrogate time series, ApEn or SampEn will be higher for the surrogate series than the original. However, is this increase in randomness (or entropy) also a reflection of increase in complexity of the representative system? No, because technically, randomization breaks the inherent structure of the originally complex series, leading to information loss, in other words loss of content/complexity [42].

In such a case, can the irregularity statistics such as ApEn and SampEn be equipped to retrieve “complexity” information as well? Well, the answer is yes, this has been made possible by the idea of “multiscaling” in entropy analysis. Costa et al. suggested that “complexity” in a system is unveiled only by exploring the system at multiple spatio-temporal scales while “irregularity” in the system can be determined from a single-scale based analysis (as is done in ApEn or SampEn). This implies: irregularity when examined at multiple spatio-temporal scales can lead us to complexity related information. Thus, Costa et al. in their work proposed to conduct SampEn estimations at multiple scales (ranging from 2 to 20), giving us the popularly known technique of “multiscale sample entropy (MSE) analysis.” MSE analysis demonstrated to detect cardiac abnormalities such as atrial fibrillation (AF) and congestive heart failure (CHF), successfully revealed complexity information from the respective HRV signals, by placing SampEn of healthy HRV above that of diseased HRV at the higher scales (beyond scale 12 for AF and scale 2 for CHF) [43]. This is owing to the fact that a healthy cardiac system is more complex in relation to one that is affected by AF or CHF [44,45].

**Figure 2:**
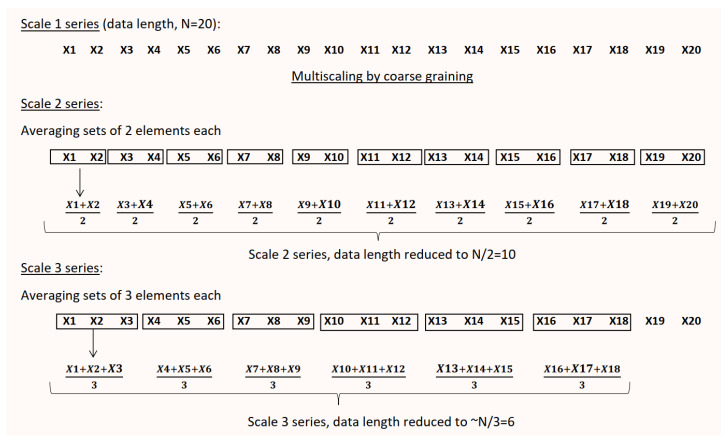
Procedure of coarse-graining in multiscale sample entropy (MSE) analysis.

The only limitation of MSE is the inability of the method to handle short-term data. At every increasing scale in MSE, the original time series is coarse-grained by the scale factor, thereby reducing data length from the original *N* to $≃\frac{N}{scale}$. The coarse-graining procedure is demonstrated in Figure 2 below.

Thus, if *N* is originally 1000, then at scale 20 the series length available for SampEn estimation will be only around 50. From the previous section on existing problems with regularity analysis, we understood that the regularity statistic SampEn cannot handle N<1000, unless the irresolute *r*-selection issue is resolved. Thus, currently the minimum length required at the highest scale in MSE is 1000. Lower the original data length *N*, lower will be the maximum scale achievable. For Costa, since she explored scales till 20, the original data length of HRV signal she used was 20,000 [43].

## 4. How Has “***r***-Dependence” Been Addressed by Other Researchers?

To rationalize *r* selection, Lake et al. [40] in 2002 formulated a generalized approach where SampEn is first calculated for a range of *r* values and then the optimal value (of *r*) is selected such that the relative error between SampEn and CP (conditional probability of a match at length m+1, given there is a match at *m* ) is minimized. The main drawback in this approach is that the initial range of *r* for which SampEn values are calculated, is again chosen from the recommended 0.1–0.25 times the standard deviation (*SD*) of the signal. This may not be ideal, since the optimal *r* value might even exist outside this range, as there is no evidence that says otherwise.

In 2008, Lu et al. [9] introduced the concept of “maximum approximate entropy (MaxApEn)” for logical selection of data specific *r*—denoted by $r_{max}$. The value of $r_{max}$ represents the tolerance at which ApEn is maximum for a given signal. Since MaxApEn was observed to correctly reflect signal irregularity, the process of selecting $r_{max}$ was considered optimal and rational [9]. However, to find $r_{max}$ and MaxApEn, ApEn corresponding to different potential *r* values must be calculated. This traditional approach is highly cumbersome [9,24] and inefficient. This involves repeated computation of ApEn for each given value of *r* ($r=\{\frac{i*SD}{100}:i\in Z\wedge 1\leq i\leq 100\})$. As a remedy, Lu et al. proposed a set of generalized equations to calculate $r_{max}$ of the signal [9]. These equations were extrapolated with reference to random Gaussian white noise signals of unit standard deviation. For this, ApEn of the signals was explored at different values of *r* between 0 and 1 varying in steps of 0.01. At a given value of *m*, $r_{max}$ was defined as a function of signal length and signal variability. In their work, Lu et al. derived generalized equations for $r_{max}$ at three different values of embedding dimension; m=2,3and4. The $r_{max}$ obtained thus was used to find MaxApEn. Despite being computationally efficient, the equation based approach has certain limitations that make its use unreliable and inconsistent: (i) since general equations were derived on the basis of random linear signals, they fail to work for signals exhibiting non-linear dynamics [24,28]; (ii) there may be applications that require the use of an embedding dimension m>4, which will then demand burdensome derivations all over again; (iii) the approach uses a partial range of *r* for calculating ApEn values, instead of finding the actual potential *r* range for the given data. It is known that a function f(x):x∈X has a real maximum at a point say $x_{max}$, if $f\left(x_{max}\right)>f\left(x\right)\forall x\in X$. Hence, knowledge of *X* in its complete form is necessary to find $x_{max}$ and $f(x_{max})$. Here, the ApEn range is restricted to values corresponding to an *r* range of [01∗SDofsignal], which may not be the required potential range to calculate $r_{max}$ and MaxApEn, (iv) the approach uses a constant *r* resolution of $\frac{1}{0.01*SD\;\mathrm{of}\;\mathrm{signal}}$ in the computation. The choice being arbitrary may not be capable of capturing all potential *r* values in the range. A low or insufficient resolution reduces the accuracy of global measures such as “maximum.” Therefore in case of calculating $r_{max}$ and MaxApEn, the entire conceivable range of *r* has to be defined with an appropriate resolution.

**Figure 3:**
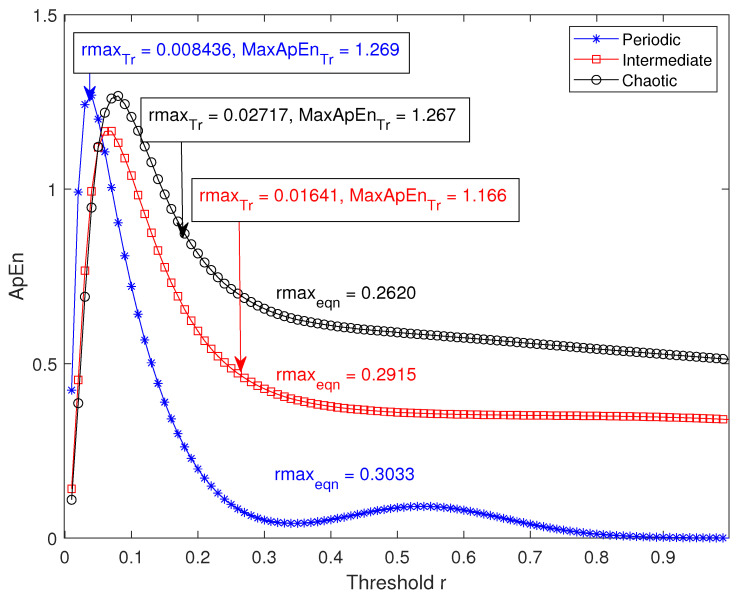
The average variations of ApEn with *r* of periodic, intermediate and chaotic data sets are shown. $r_{max_{Tr}},MaxApEn_{Tr}$ values of the data calculated by the traditional method are indicated in the top left corner and $r_{max_{Eqn}}$ values calculated by the equation method are marked close to their respective curves.

To demonstrate this, the average variation of ApEn with *r* ($r=\{\frac{i*SD}{100}:i\in Z\wedge 1\leq i\leq 100\})$ for three types of data (periodic, intermediate and chaotic) has been plotted in Figure 3. Ten realizations each of the three sets (periodic, intermediate and chaotic) of signals are used here. The logistic map equation from chaotic systems toolbox of MATLAB 2019Rb is used: $x_{n+1}=ax_{n}\left(1-x_{n}\right)$; *a* denotes level of irregularity; $x_{n}$ is the initial value and is kept at 0.5. The generated signal is then superimposed with a random white noise whose noise level is kept at 0.1. *a* is equal to 3.5, 3.75 and 4 in the function corresponding to a periodic, intermediate and chaotic level signal respectively.

The average $r_{max_{Tr}}$($r_{max}$ obtained by the traditional approach) in each of the three cases is found to be 0.008436, 0.01641 and 0.02717 respectively. On the other hand, the average $r_{max_{Eqn}}$($r_{max}$ obtained by Lu et al.’s approach ) value for each of the three data sets is 0.3033, 0.2915 and 0.2620 respectively (Figure 3). For periodic and intermediate signals, the average $r_{max_{Eqn}}$ obtained by Lu et al. method [9] is greater than 1∗SD of the signal, which is non-existent in the defined $ApEn_{Tr}$ (ApEn profile obtained traditionally in the *r* range of 0.01*SD* to 1*SD*) space. Moreover, the average $MaxApEn_{Tr}$ (MaxApEn obtained by the traditional approach) values for periodic and intermediate signals are 1.269 and 1.166 respectively, whereas for the same signals $MaxApEn_{Eqn}$ (MaxApEn obtained by Lu et al.’s approach) values are non-existent in the defined $ApEn_{Tr}$ space. For the chaotic data, although $r_{max_{Eqn}}$ value is found within 1∗SD of the signal (Figure 3), both $r_{max_{Tr}}$(0.02717) and $MaxApEn_{Tr}$ (1.267) values are significantly different from $r_{max_{Eqn}}$(0.2620) and $MaxApEn_{Eqn}$ (0.55). It is obvious that, the $MaxApEn_{Eqn}$ value obtained for chaotic signal is smaller than the actual maximum ApEn value of the signal, which is measured as $MaxApEn_{Tr}$.

In 2009, a new entropy measure called FuzzyEn (fuzzy entropy) was introduced by Chen et al. [8], where the tolerance parameter *r* was replaced by a tolerance function (a fuzzy function involving *r*), to decide sub-sequence matching. The method was a considerable improvement from ApEn and SampEn, since the estimate’s dependence on *r* significantly reduced.

### 4.1. Fuzzy Entropy (FuzzyEn)

For a given signal of length *N*, FuzzyEn is given by

(12) $$\begin{array}{cc} FuzzyEn(N,m,r) & =\ln\frac{\Phi^{m}\left(r\right)}{\Phi^{m+1}\left(r\right)} \end{array}$$

where

(13) $$\begin{array}{cc} \Phi^{m}\left(r\right) & =\frac{1}{N-m}\begin{array}{c} N-m\\ \sum \\ i=1 \end{array}C_{i}^{m}\left(r\right) \end{array}$$

and

(14) $$\begin{array}{cc} C_{i}^{m}\left(r\right) & =\frac{1}{N-m-1}\begin{array}{c} N-m\\ \sum \\ i=1,i\neq j \end{array}D_{ij}^{m} \end{array}$$

Here, $D_{ij}^{m}=\mu \left(d_{ij}^{m},r\right)$ is the similarity degree defined by a fuzzy membership function μ which in this case is an exponential function given by $\exp(-{\left(\frac{d_{ij}}{r}\right)}^{2})$. Thus, $C_{i}^{m}\left(r\right)$ is the probability of a vector $X_{j}^{m}$ to be similar to the vector $X_{i}^{m}$ by a degree $D_{ij}^{m}$, for 1≤j≤(N−m),j≠i. For all experiments conducted in this study, FuzzyEn is evaluated at an *r* value of 0.15∗SD of signal and an *m* value of 2.However, FuzzyEn was still bound by limitations of having to make an irrational assumption of *r* [30]. Liu et al. [46] in 2013, proposed FuzzyMEn (fuzzy measure entropy), to improve the stability (with respect to *r*) and discriminating ability of FuzzyEn. More recently, in 2015, Ji et al. [47] have yet again tried to improve the stability, discriminating ability and noise robustness of FuzzyEn, by introducing rFuzzyEn (refined fuzzy entropy). Despite the advent of several versions of FuzzyEn, the fundamental problem of irresolute *r* selection remains unsolved.

Lake et al. [31] introduced a new regularity measure called CosEn (Coefficient of SampEn) in 2011. Here, the optimal value of threshold *r* is chosen based on the principle of a “minimum numerator count.” This method eliminates the data length limitation seen in SampEn estimation, thereby enabling analysis on signals with *N* as low as 12 data points. In 2018, another study [48] implemented the same “minimum numerator count” concept on FuzzyMEn (fuzzy measure entropy) and achieved better efficiency than CosEn. However, the problem with “minimum numerator count” method is that it needs the user to keep changing *r* value, and repeatedly carry out the estimation procedure till an optimal CP (conditional probability of a match at length m+1, given there is a match at *m* ) is reached. Thus, the method undoubtedly has disadvantages discussed above, as in the earlier approaches.

### 4.2. Normalized Fuzzy Measure Entropy (Normalized FuzzyMEn)

From the given time series, a set each of local and global vector sequences are formed (as elaborated in [32]), $L_{i}^{m}$ and $G_{i}^{m}$ respectively at an embedding dimension *m*. Then, the local and global similarity degrees or fuzzy functions are computed as

(15) $$DL_{ij}^{m}\left(n_{L,}r_{L}\right)=\exp\left(-{\left(\frac{dL_{ij}^{m}}{r_{L}}\right)}^{n_{L}}\right)$$

and

(16) $$DG_{ij}^{m}\left(n_{G,}r_{G}\right)=\exp\left(-{\left(\frac{dG_{ij}^{m}}{r_{G}}\right)}^{n_{G}}\right)$$

where $dL_{ij}^{m}$ and $dG_{ij}^{m}$ are the distances between the local and global vector sequences respectively, computed as per [32]. The mean values of these fuzzy functions are computed as $BL^{m}\left(n_{L},r_{L}\right)$ and $BG^{m}\left(n_{G},r_{G}\right)$, respectively. The same steps when repeated with an embedding dimension m+1, produce mean fuzzy functions $AL^{m+1}\left(n_{L},r_{L}\right)$ and $AG^{m+1}\left(n_{G},r_{G}\right)$. The fuzzy local and global measure entropies are then estimated as

(17) $$\begin{array}{c} FuzzyLMEn=\\ \ln\left(\frac{BL^{m}\left(n_{L},r_{L}\right)}{AL^{m+1}\left(n_{L},r_{L}\right)}\right)+\ln\left(2r_{L}\right)-\ln\left(RR_{mean}\right) \end{array}$$

and

(18) $$\begin{array}{c} FuzzyGMEn=\\ \ln\left(\frac{BG^{m}\left(n_{G},r_{G}\right)}{AG^{m+1}\left(n_{G},r_{G}\right)}\right)+\ln\left(2r_{G}\right)-\ln\left(RR_{mean}\right) \end{array}$$

respectively. Finally, normalized FuzzyMEn is given by

(19) $$H_{NF}=FuzzyLMEn+FuzzyGMEn$$

Here, $r_{L}$ and $r_{G}$ are the local and global thresholds of distance and are estimated using the minimum numerator count method [31,32]. $n_{L}$ and $n_{G}$, the local and global similarity weights both are assigned a value of 2 here.

In 2015, there was yet another attempt to address *r*-selection issues in entropy estimates. Peng Li came up with DistEn (distribution entropy) [10], where the tolerance parameter *r* was completely eliminated and replaced by another parameter “*M*.” Now, DistEn is an approach based on empirical distribution functions and *M* constitutes the number of bins in this distribution. DistEn does compute vector-to-vector distances as in the case of statistics such as SampEn and FuzzyEn, however instead of then finding vector matches (using *r*), DistEn generates a probability distribution function of these distances (using *M*), eventually calculating the Shannon entropy. Despite the fact that DistEn performs far better than SampEn (specially at lower data lengths), it is to be understood that they do not come from the same family of probability measurement. Empirically, SampEn of a signal is a measure of its conditional probability to remain similar at embedding dimensions *m* and m+1. Thus, signal regularity is measured at embedding dimensions *m* and m+1. On the other hand, for DistEn, entropy is measured from the distribution of vector distances only at an embedding dimension *m*. Hence, the information obtained using these two measures are entirely different and so cannot be taken as a direct answer to the problem of irresolute *r* selection in SampEn (or any of the KS-entropy methods).

Thus, as far as HRV regularity analysis is concerned, parametric dependence on *r* is still a huge existing problem. A solution to this could result in ApEn, SampEn, FuzzyEn and the like being made suitable, more accurate and reliable for “short-length” (N<1000, i.e., less than 15 min data) HRV irregularity and complexity analysis.

## 5. Entropy Profiling as a Solution

Most existing entropy methods are highly dependant on a single choice of the tolerance parameter (*r*) for measuring signal irregularity or complexity. This single choice of *r* is usually picked from a predefined range of values. When it comes to the analysis of short-term signals, these methods become all the more sensitive to this choice of *r*. If not chosen rightly, the estimation might end up giving us inaccurate information about the signal. In order to improve the accuracy and reliability of signal information retrieved, we hypothesize that it is necessary to find entropy at all potential *r* values, instead of a single chosen *r* value. This approach of entropy profiling (in contrast to entropy estimation) will give a more complete information as against the partial one obtained when using just a single *r*. Now, it becomes equally important to demonstrate accuracy in finding all potential *r* values for such a profiling. For this, we introduce a data-driven algorithm that finds potential *r* values by exploiting the dynamics of the given signal [49,50]. The algorithm is automatic and not based on any random assumption as in other methods. In the following two subsections, we explain the need for entropy profiling in the context of measuring signal irregularity and signal complexity, respectively.

### 5.1. Signal Irregularity

Approximate entropy (ApEn), introduced by Pincus in 1991 [13] was the first in series from the Kolmogorov–Sinai (KS) entropy family to be widely known as a useful non-linear measure for short-term HRV analysis [14,25,26,51,52,53,54]. ApEn is an approximate quantification of KS entropy [14]. ApEn is a function of data length *N*, embedding dimension *m* and tolerance of inter-vector distance *r*. ApEn measures the conditional probability of two segments of a signal (of length *N*) matching at a length m+1 if they match at a length *m*. Segment matching is evaluated with the help of the parameter *r*, where r=k∗SDofsignal; *k* takes values between 0 and 1 [13,14]. SD denotes standard deviation of the time-series signal. ApEn that is considered a biased regularity measure was later in 2000 replaced by sample entropy (SampEn) [16] and in 2007 replaced by corrected approximate entropy (CApEn) [55]. The source of the bias was the counting of self-matches in signal segments. Additionally, the estimation of ApEn was found to be inconsistent with change in parameters *m* and *r*, causing flipping of values [16,26,53]. CApEn corrected ApEn’s bias towards regularity by eliminating self-match counts from the algorithm [55]. However, CApEn did not gain much popularity within the research community due to the already well accepted SampEn. In addition to eliminating self-match counts from its formulation, SampEn shows a more consistent behavior in situations where ApEn does not [16]. However, SampEn also has its limitations. Relative consistency of SampEn is not exhibited with all data sets. Particularly, for data where N<200, SampEn is as sensitive to the change of parameters *m* and *r* as ApEn [16,26]. The tolerance of segment-match *r* is found to be the most critical of parameters. Parametric sensitivity of ApEn and SampEn to “*r*” does negatively impact the proficiency of signal information retrieval. A perfect choice of *r* is therefore most essential to produce accurate values of SampEn [9,25,26].

As mentioned earlier, traditional SampEn measurement uses a single *r* choice for its estimation. This approach is unreliable mainly because of SampEn’s sensitivity to any change in *r*. For the same signal, a different choice of *r* produces a different SampEn [16], meaning that the signal could have two estimates for the same information. This will be completely misleading. Consequently, we believe that generating a complete SampEn profile in response to a data specific set of *r* values will address the issue. Here, we will be able to use even the insensitivity of SampEn as added information, thereby turning the limitation into an advantage. Such an approach will lead to enhanced information retrieval from short-term HRV signals.

Given a signal, there are two challenges in trying to acquire a potential set of all *r* values:

1. Insufficient information: It is first of all necessary to know how many *r* values should be in the set, what should be the minimum and maximum values of *r* in the set and so on. In other words, the range and resolution of *r* values in the set should be defined. We know that previous studies have used a general *r* range of (0−1)∗SD of signal and highest *r* resolution of $\frac{1}{0.1*SD\;\mathrm{of}\;\mathrm{signal}}$ for the purpose. However, these recommended values do not hold any proof of uniform credibility across all data sets [9,18,25,28]. Moreover, these choices have mostly been random. Since our approach aims at generating a complete profile for a given signal, we must ensure that all significant changes in *r* and thereby entropy are captured. This cannot be achieved if we go with random parametric selection once again. Data driven choices become indispensable here.
2. Computational expense involved: Even if we are successful in listing all potential *r* values for the given signal, the next challenge is to estimate entropy at each of these *r* values. With available expertise, the only way to do this is by repetitively estimating entropy at each *r*. The process could be complex and time consuming for high dimensional signals. A computationally efficient approach also becomes indispensable here.

This is where our novel “cumulative histogram method (CHM)” for entropy profiling finds place, the method being computationally efficient and completely data driven [49,50].

### 5.2. Signal Complexity

Chaos or order in a physiological system does not always reflect its level of complexity [19,43,56]. A system with highly uncorrelated interactions will be more complex to comprehend. A signal arising from such a complex system should be categorized as complex, irrespective of the regularity of patterns seen in it [56]. For instance, arrhythmia is a condition where there is additional irregularity in the normal heart rate pattern. However, a normal cardiac system has better reflexes and adaptability to a changing environment than an arrhythmic cardiac system, making it a more complex system [43]. Thus, an arrhythmic signal can be more irregular than a normal heart signal, despite coming from a low complex system. In such cases, it becomes necessary to particularly extract complexity information (not just irregularity) from the signal. For this, the signal must be examined across various temporal and spatial scales, which is equivalent to exploring the structural complexity of the system.

ApEn and SampEn are signal irregularity measures and do not give any inherent information about system complexity [16,56,57]. Multiscale sample entropy (MSE) was proposed in 2002 [19] to quantify a system’s complexity-based information. Here, SampEn was estimated at multiple temporal scales by using a coarse graining procedure of the original time series. Consequently, irregularity measured at higher temporal scales has the potential to reveal complexity-based information. Signals associated with cardiac pathologies such as atrial fibrillation showed higher values of ApEn and SampEn, compared to those of healthy cardiac signals [19,43,44,45,58,59]. It must be noted that a healthy cardiac system is much more complex in nature than the pathological ones. Thus, the higher values of entropy for pathological signals do not reflect their system’s complexity, but the opposite.

MSE employs a coarse graining procedure, where as the scale factor increases, original data length keeps reducing by the factor. Additionally, since SampEn has its own limitations in handling short-term data, MSE works accurately only for relatively longer lengths of data. The original data length is required to be high enough so that even at the highest scale factor used in MSE, the resultant coarse-grained signal (with reduced data length) is fit to be handled by SampEn. On the other hand, researchers have also tried enabling MSE to analyze short-length signals. A few modifications to MSE analysis such as modified MSE (MMSE), short-time MSE (sMSE) and refined generalized MSE ($RMSE_{\sigma^{2}}$) demonstrated the use of MSE on short-term synthetic data [48,60,61]. Their use on physiologic signals have not been explored yet.

Our proposed method of “entropy profiling” breaks all constraints and equips SampEn to handle short-length heart rate data. Thus, we hypothesize that augmenting the multiscaling procedure with “entropy profiling” will eliminate the former’s dependency on long-term data [20].

### 5.3. Formulation of Entropy Profiling

Geometric interpretations of entropy profiling [49]:

We use the cumulative histogram method (CHM) to generate a SampEn profile. The method uses a data-driven approach of multiresolution binning for acquiring the potential set of all *r* values. This *r* set is then used to build the entropy profile. The steps are listed and illustrated below.

**Figure 4:**
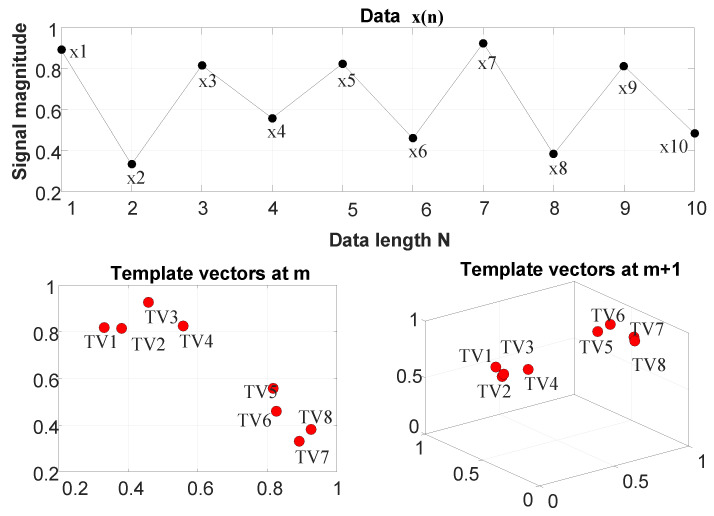
**Upper** panel: sample data x(n) of data length *N*. **Lower** panels: generation of template vectors at embedding dimensions *m* and m+1. Here, N=10 and m=2. (Figure adapted from [49]).

Let a time series of length *N* be defined as $\left\{x(n):1\leq n\leq N\right\}$. An example data of length N=10 has been shown in Figure 4.

**Figure 5:**
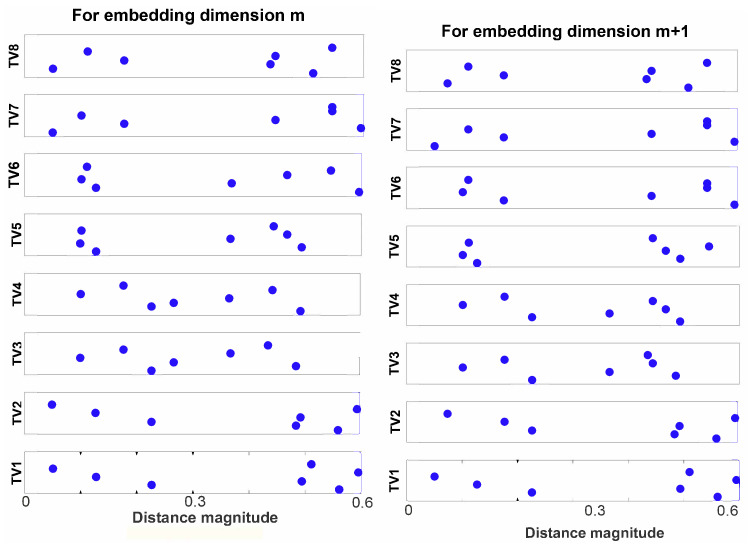
Illustration of step 3 of CHM: estimation of vector distances at embedding dimensions *m* and m+1 of each template vector from other vectors. **Left** panel: elements of the matrix $d^{m}$. **Right** panel: elements of the matrix $d^{m+1}$. (Figure adapted from [49]).

**Figure 6:**
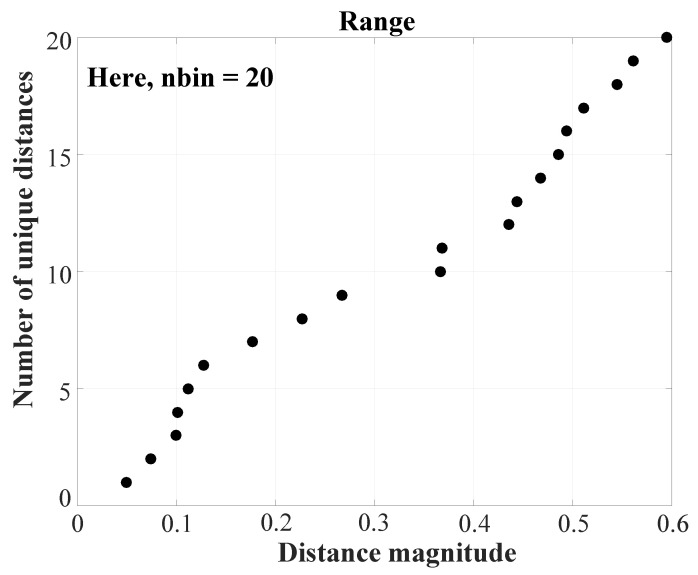
Illustration of step 4 of CHM: range, the set of unique elements of *D*, sorted in ascending order. *D* is the matrix combining elements of $d^{m}$ and $d^{m+1}$. $n_{bin}$ is the number of elements in range. (Figure adapted from [49]).

**Figure 7:**
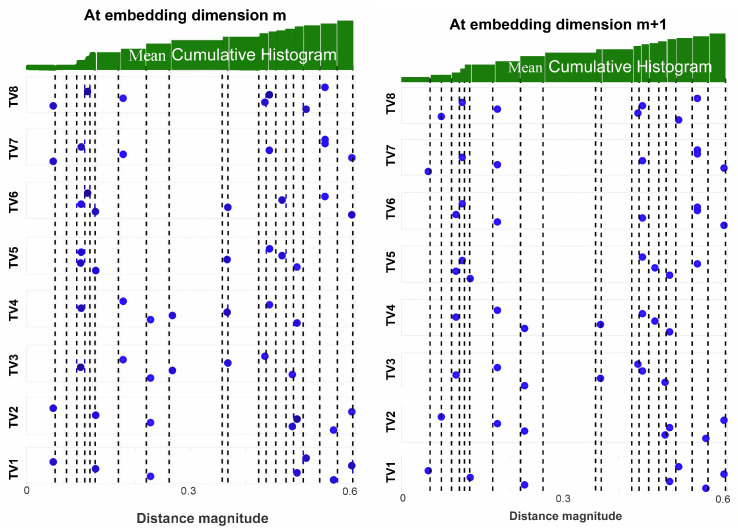
Illustration of steps 6 and 7 of CHM: estimation of the cumulative distribution functions. **Left** panel: $cdf^{m}$ at embedding dimension *m*. **Right** panel: $cdf^{m+1}$ at embedding dimension m+1. (Figure adapted from [49]).

**Figure 8:**
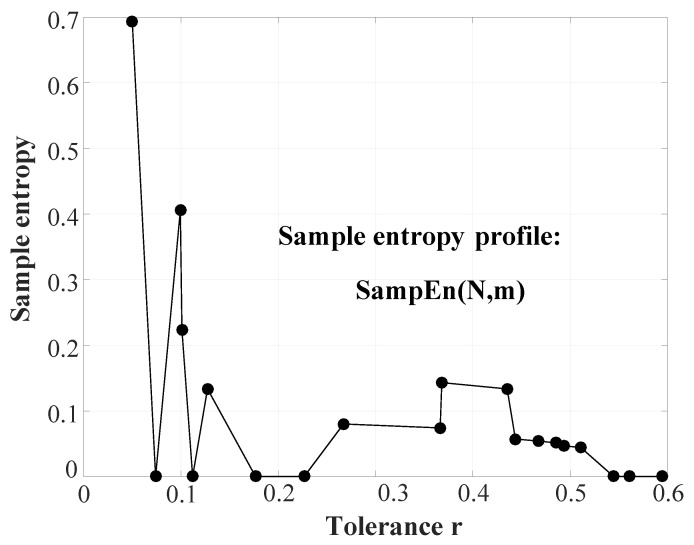
Illustration of step 9 of CHM: Generation of the SampEn profile. The number of SampEn values (also the number of *r* values) equals $n_{bin}$. (Figure adapted from [49]).

For a given value of the embedding dimension *m*:

1. Form (N−m) vectors of length *m* each, given by
  $$\left\{X_{i}^{m}:1\leq i\leq \left(N-m\right)\right\}\mathrm{where}$$
  (20) $$X_{i}^{m}=\left\{x(i+k):0\leq k\leq m-1\right\}$$
2. Similarly, form (N−m) vectors of length (m+1) each, given by
  $$\left\{X_{i}^{m+1}:1\leq i\leq \left(N-m\right)\right\}$$
  (21) $$\mathrm{where},\;X_{i}^{m+1}=\left\{x(i+k):0\leq k\leq m\right\}$$
  The above two steps have been illustrated in the lower sub panels of Figure 4 for an m=2.
3. Take each $X_{i}^{m}$ vector of step 1 as a template vector and find its distance from every vector $X_{j}^{m}$, where the distance is given by
  (22) $$\begin{array}{cc} d_{ij}^{m} & =\{\max|X_{i}^{m}-X_{j}^{m}\left|:\;1\leq j\leq (N-m),j\neq i\right\}, \end{array}$$
  so for an *i*-th template vector, the distance vector for an embedding dimension *m* will be of the following form
  (23) $$d_{i}^{m}=d_{ij}^{m}:1\leq j\leq \left(N-m\right),j\neq i$$
  similarly, for an embedding dimension m+1, the distance vector can be obtained as
  (24) $$d_{i}^{m+1}=d_{ij}^{m+1}:1\leq j\leq \left(N-m\right),j\neq i$$
  This step has been illustrated in Figure 5.
4. Let *D* be the matrix containing all elements of $d^{m}$ and $d^{m+1}$. Then, we define range as the set of all unique elements of *D*, sorted in ascending order. Additionally, let $n_{bin}$ be the number of elements in range (shown in Figure 6).
5. From the distance vector $d_{i}^{m}$, the cumulative distribution function $cdf_{i}^{m}$ is calculated as:
  (25) $$cdf_{i}^{m}\left(q\right)=p\left(d_{i}^{m}\leq range\left(q\right)\right),\;\mathrm{for}\;1\leq q\leq n_{bin}$$
  where *p* is the probability.
6. Repeat step 5 for all $d_{i}^{m}$:1≤i≤(N−m) to calculate the entire cumulative distribution matrix $cdf^{m}$ for the embedding dimension *m* as shown:
  (26) $$cdf^{m}=\left[\begin{array}{ccc} cdf_{1}^{m}\left(1\right) & \cdots & cdf_{1}^{m}\left(n_{bin}\right)\\ cdf_{2}^{m}\left(1\right) & \cdots & cdf_{2}^{m}\left(n_{bin}\right)\\ \vdots & \cdots & \vdots \\ cdf_{N-m}^{m}\left(1\right) & \cdots & cdf_{N-m}^{m}\left(n_{bin}\right) \end{array}\right]$$
  (shown in left sub panel of Figure 7).
7. Similarly for embedding dimension m+1 the cumulative distribution matrix $cdf^{m+1}$ can be obtained by repeating step 4 for all $d_{i}^{m+1}$.
  (27) $$cdf^{m+1}=\left[\begin{array}{ccc} cdf_{1}^{m+1}\left(1\right) & \cdots & cdf_{1}^{m+1}\left(n_{bin}\right)\\ cdf_{2}^{m+1}\left(1\right) & \cdots & cdf_{2}^{m+1}\left(n_{bin}\right)\\ \vdots & \cdots & \vdots \\ cdf_{N-m}^{m+1}\left(1\right) & \cdots & cdf_{N-m}^{m+1}\left(n_{bin}\right) \end{array}\right]$$
  (shown in right sub panel of Figure 7).
8. Now, for each column of $cdf^{m}$ and $cdf^{m+1}$, we calculate the average of the probabilities, which is represented as $\theta^{m}\left(q\right)$ and $\theta^{m+1}\left(q\right)$ respectively.
  (28) $$\theta^{m}\left(q\right)=\frac{1}{N-m}\begin{array}{c} N-m\\ \sum \\ i=1 \end{array}\left(cdf_{iq}^{m}\right),1\leq q\leq n_{bin}$$
  (29) $$\theta^{m+1}\left(q\right)=\frac{1}{N-m}\begin{array}{c} N-m\\ \sum \\ i=1 \end{array}\left(cdf_{iq}^{m+1}\right),1\leq q\leq n_{bin}$$
9. SampEn which is an approximation of the conditional probability of two segments matching at a length m+1 if they match at *m*, can be defined from $\theta^{m}$ and $\theta^{m+1}$ as $SampEn=\ln\frac{\theta^{m}}{\theta^{m+1}}$. In this case, we will have $n_{bin}$ number of SampEn values since $\theta^{m}$ and $\theta^{m+1}$ are calculated over $1\leq q\leq n_{bin}$ (shown in Figure 8). This can be expressed as $SampEn\left(q\right)=\ln\frac{\theta^{m}\left(q\right)}{\theta^{m+1}\left(q\right)}$, where $1\leq q\leq n_{bin}$.

It must be noted here that each value of *q* is in fact a unique value of *r*. Thus, there will be $n_{bin}$ number of unique *r* values used for entropy profiling. The algorithm automatically selects the value of $n_{bin}$, derived from the dynamics of the signal. Here, $n_{bin}$ is a data-driven value (covering all potential *r* values for the given signal) and hence the best choice to generate a complete entropy profile. This is unlike other methods where the choice of $n_{bin}$ is either random [10,62] or logically selected [63].

CHM performs SampEn profiling with the aid of simplified matrix operations and not looped iterations as in traditional estimations. Moreover, the range and resolution of *r* are not selected arbitrarily, but derived from the dynamics of the given data. It is to be noted that the *r* resolution used here is not a single constant value. Based on the change of inter-vectors distances, the *r* value takes up a multiresolution spread. This can be called a multiresolution binning scheme.

### 5.4. New Measures of Irregularity from a SampEn Profile

Total sample entropy (TotalSampEn):

TotalSampEn is the summation of all values in the SampEn profile of a signal.

(30) $$TotalSampEn=\sum_{q=1}^{n_{bin}}SampEn\left(q\right)$$

The value is usually very high because of the summing effect. The high value of TotalSampEn can be normalized by dividing it by the respective number of bins in each case. This gives rise to another measure called the average sample entropy or AvgSampEn.

Average of sample entropy (AvgSampEn:)

(31) $$AvgSampEn=\frac{1}{n_{bin}}\sum_{q=1}^{n_{bin}}SampEn\left(q\right)$$

Extracting new measures from an entropy profile is not restricted to TotalSampEn and AvgSampEn. Various other forms of information can be extracted from the complete entropy profile [49,50,64,65].

## 6. Case Studies

The various statistics used for study in our results section include SampEn, FuzzyEn, normalized FuzzyMEn, TotalSampEn and AvgSampEn that are represented as $H_{S}$, $H_{F}$, $H_{NF}$, $H_{TS}$ and $H_{AS}$ respectively in the upcoming tables and figures.

### 6.1. Age Based HRV Classification

Data used: HRV signals were obtained from the Fantasia module of the PhysioNet database [66]. The subject population included forty healthy adults belonging to two different age groups: (1) “young,” 21–34 years; and (2) “elderly,” 68–85 years. Both age groups contained twenty subjects each, having an equal number of men and women. HRV data was extracted from a 120 minutes recording of an individual subject’s electrocardiogram (ECG), sampled at a frequency of 250Hz. An automated RR interval detection algorithm was then used to obtain the HRV of subjects [67]. Each HRV signal is selected from the beginning to lengths of 50, 100, 150, 200, 250 and 300 beats.

**Figure 9:**
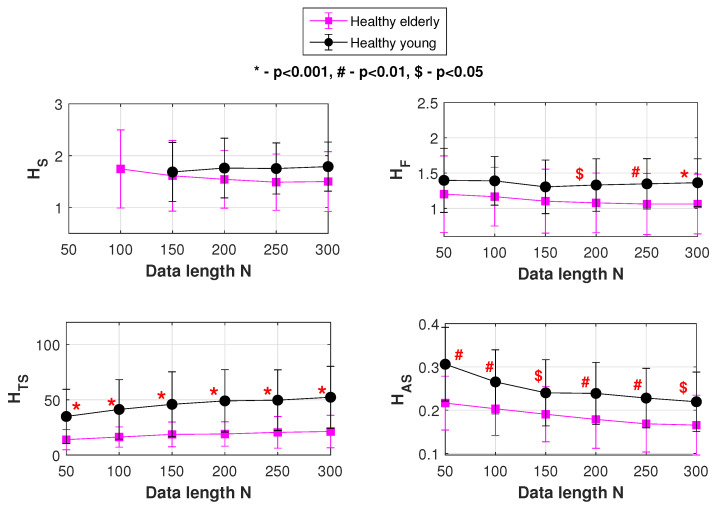
Mean and *SD* variations of $H_{S}$, $H_{F}$, $H_{TS}$ and $H_{AS}$. Classification of the “elderly” and “young” groups of healthy heart rate variability (HRV) data.

For the the “young” and “elderly” groups of HRV data, plots showing the mean and *SD* variation of $H_{S}$, $H_{F}$, $H_{TS}$ and $H_{AS}$ are shown in Figure 9. In the figure, at each of the data lengths used, the performance (statistical significance) of classification is indicated by *p* values. *p* value is the probability of samples of one population being equal to or greater than samples of another population and is obtained using Mann-Whitney U test. *p* can take values between 0 and 1. In this study, p<0.05 is considered statistically significant.

Table 1 summarizes the results of Figure 9, and lists another statistical estimate of performance efficiency—the AUC (area under the ROC curve). AUC is the probability of a classifier to rank a randomly chosen instance *X* higher than a randomly chosen instance *Y*. *X* and *Y* denote samples from two independent populations. An AUC of 0.5 means the distribution of features is similar in the two groups with, while an AUC of 1.0 indicates that the distribution of features in the two groups are completely dissimilar.

Table 1 compares the performances of $SampEn(H_{S})$, $FuzzyEn(H_{F})$ and our entropy profile measures $TotalSampEn(H_{TS})$ and $AvgSampEn(H_{AS})$ in classifying the age based HRV data. At all data lengths, the *p* value for SampEn is either insignificant or inapplicable (due to an indefinite SampEn value. This is an inherent problem that happens with some data due to SampEn’s formulation and random *r* selection).

With FuzzyEn, significance in data classification begins only after a data length of 150. In the case of our entropy profile-based TotalSampEn and AvgSampEn, data classification is statistically significant at all data lengths. Where traditional estimation methods such as SampEn and FuzzyEn fall back, TotalSampEn and AvgSampEn prove efficient.

AUC values of Table 1 also support our observations made so far. At the lower data lengths (N<200), data classification is done most efficiently by TotalSampEn. AvgSampEn and FuzzyEn follow suit. However, SampEn is incapable of classifying the age based population at such low data lengths. At the higher data lengths, there is a small improvement in the case of SampEn. However, compared to the other three measures at the same point, SampEn still falls behind. At the higher data lengths, it is TotalSampEn that performs the best. AvgSampEn and FuzzyEn show almost similar performances at this stage. It can be unambiguously seen that at both the lower and higher data lengths, entropy profiling-based measures such as TotalSampEn and AvgSampEn are the ones that offer superior data classification of the age based HRV groups.

### 6.2. Disease Based HRV Classification

HRV signals were obtained from the MIT-BIH module of the PhysioNet database [66]. The subject population included 66 adults belonging to two groups: (1) “healthy” cardiac state and (2) “arrhythmic” cardiac state. The healthy group contained 18 subjects, with 5 men, aged 26 to 45, and 13 women, aged 20 to 50. The arrhythmic group had 48 subjects [68]: 25 men aged 32 to 89 years, and 22 women aged 23 to 89 years. Each HRV signal is selected from the beginning to lengths of 50, 100, 150, 200, 250 and 300.

**Figure 10:**
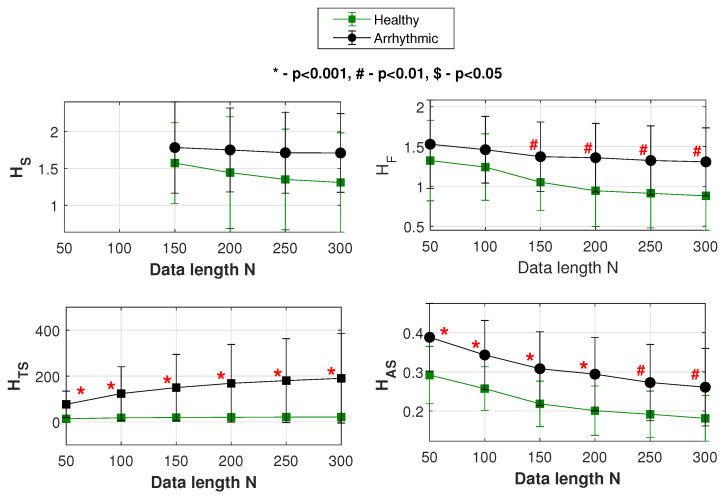
Mean and *SD* variations of $H_{S}$, $H_{F}$, $H_{TS}$ and $H_{AS}$. Classification of the “healthy” and “arrhythmic” groups of HRV data.

For the above signals, plots showing the mean and *SD* variation of $H_{S}$, $H_{F}$, $H_{TS}$ and $H_{AS}$ are shown in Figure 10. In the figure, at each of the data lengths used, the performance (statistical significance) of classification is indicated by *p* values. Table 2 summarizes the results of Figure 10 and lists the respective AUC values as well.

Table 2 compares the performances of $SampEn(H_{S})$, $FuzzyEn(H_{F})$ and our entropy profile measures $TotalSampEn(H_{TS})$ and $AvgSampEn(H_{AS})$ in classifying the healthy from diseased HRV data.

Looking at the *p* values, here also SampEn has an insignificant or indefinite performance at all the data lengths. With FuzzyEn, a significant data classification happens for N>100. On the other hand, at all data lengths TotalSampEn and AvgSampEn give significant performances classifying the data.

Looking at the AUC values, we see that there is again an improvement in SampEn performance as N increases, though incomparable to the other measures. Irrespective of data length, it is the entropy profile-based measures that display a consistently superior performance in classifying healthy from arrhythmic HRV data.

### 6.3. HRV Complexity Analysis

HRV data belonging to two groups namely (1) “healthy” cardiac state and (2) “atrial fibrillated (AF)” cardiac state, were obtained from the MIT-BIH module of the PhysioNet database [66]. The healthy group contained 18 subjects: 5 men, aged 26 to 45, and 13 women, aged 20 to 50. The AF group had 25 subjects [68], all with mostly paroxysmal atrial fibrillation. All signals have been manually filtered for the removal of ectopic beats.

As mentioned in Section 5.2, the closest work that has tried HRV complexity analysis using short-term signals has used normalized FuzzyMEn. We thereby compare the efficiency of entropy profiling with that of multiscale SampEn and normalized FuzzyMEn.

**Figure 11:**
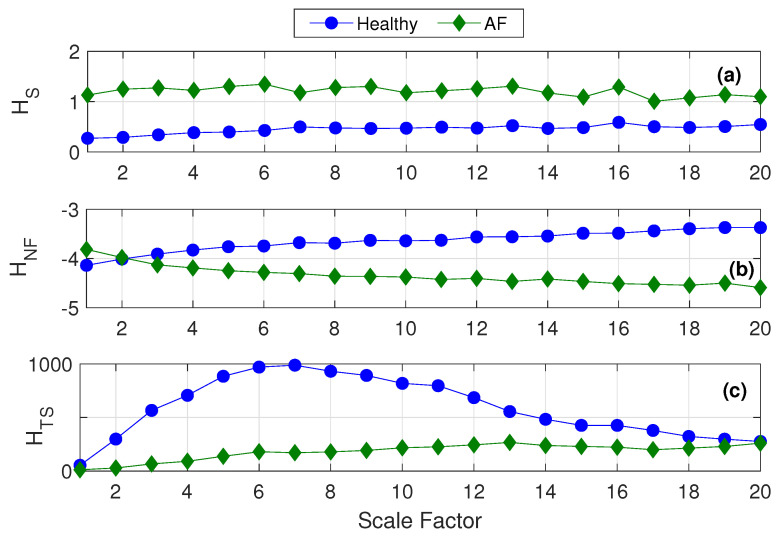
Multi scale entropy analysis of healthy and atrial fibrillation HRV data using (**a**) SampEn, (**b**) normalized FuzzyMEn and (**c**) TotalSampEn. Here data length N=1000 [20].MSE analysis of RR-interval time series from healthy and atrial fibrillation subjects

For the data length N=1000, we estimate the respective multiscaled versions of $H_{S}$, $H_{NF}$ and $H_{TS}$ using scales 1 to 20. Figure 11a proves that multiscaling on SampEn incorrectly places entropy of healthy signals at a lower value than that of the diseased ones. Panels (b) and (c) of Figure 11 show that multiscaling on $H_{NF}$ and $H_{TS}$ works in favor of a better complexity analysis since the entropy levels of the more complex healthy signals are rightly placed higher to those of the less complex diseased ones.

## 7. Discussion

Heart rate variability is a useful diagnostic indicator of physiology and pathology in the human system. Several forms of information can be derived from a HRV signal for diagnostic and analytic purposes. Simple statistical or geometrical methods to more complex nonlinear options are available for extracting useful HRV information. The information retrieval becomes challenging as data length decreases. For short recordings of HRV, getting accurate and reliable information is not easy. At the same time, short-term HRV signals are preferred over long-term counterparts, since the short-term ones are easier, less expensive and more patient-friendly to obtain and process. KS-entropy algorithms such as ApEn and SampEn are the most popularly used in this regard. These methods assess irregularity or complexity information from a given HRV signal, by detecting patterns and pattern matching in the signal. A typical KS-entropy algorithm finds the conditional probability of signal segments to match at a length m+1 if they match at a length *m*, the original signal length being *N* and the threshold of match being *r*.

Despite the good reputation of entropy methods to handle short-term HRV signals, they are widely known to be affected by parametric dominance. This is especially true when it comes to the tolerance parameter *r*. Entropy formulations require the selection of an appropriate value for parameter *r*, given a signal of length *N*. The choice of *r* is generally made from a predefined set of values, commonly used in HRV analysis. Several studies have reported a lack of logical clarity in making such a choice in addition to cases where these choices fail. A wrong choice of *r* is capable of generating completely wrong estimates of entropy, giving erroneous HRV information.

The most appropriate choice of *r* for a given signal depends purely on the nature of the signal, e.g., the signal dynamics and signal length. It is said that *r* being too small or too large has biased impacts on information retrieval. A high or low value of *r* is a very relative term, since we do not have a method to decide the perfect *r* for a specific signal. Having said that, rather than randomly selecting a single *r* and allowing entropy to give us partial information (can be biased or misleading), we believe that it is an advancement to capture the entire domain of entropy, in response to variations in *r*.

Our novel idea of entropy profiling was introduced in the year 2016 [50] to facilitate the generation of an entire profile of entropy based information, rather than a single-pointed one. The major impacts created by our method are listed below:

1. Reduced parametric dependence:
  The idea of entropy profiling with respect to the parameter *r* makes entropy a function of just *N* and *m*. *r* is completely eliminated from the list of input parameters that need the user’s discretion to be chosen. This is in contrast to the traditional case where entropy is a function of N,m and *r*. In short, entropy(N,m,r) now becomes entropy(N,m). Thus, parametric dependence of traditional formulations is significantly reduced. The CHM algorithm used for *r*-based entropy profiling is completely data driven. It automatically computes all potential *r* values for the given data. This relieves the user from having to choose a logical or appropriate value for *r* to be used, as in the case of traditional entropy estimation.
2. Short-term analysis and enhanced information retrieval:
  An irresolute *r* choice has placed its share of limitations over the use of data length *N*. With the available generalized mode of *r*-selection, entropy algorithms could be used only on data lengths N≥1000—this being approximately 15 min of HRV data, is surely considered short-term data. However, the inability of these algorithms to work on N<1000 (highly preferred for ease of data acquisition) is majorly due to lameness in *r*-selection scheme, which can therefore surely be rectified. By generating a complete set of *r* values and thereby all possible entropy estimates specific to the given signal (of any *N*), CHM does not place any kind of restrictions on data length. With a signal of any given *N*, CHM unconditionally tries and retrieves as much information from it as possible.The novel approach of entropy profiling is superior to traditional estimations as: (i) it eliminates the ambiguity that existed in choosing the right value of *r* for entropy estimations and (ii) it enhances the quality and reliability of signal information retrieved, specially from a short-length signal.
  An entropy profile contains accurate and complete information about signal irregularity. Different forms of the information can be derived from the generated profile and are called secondary measures of irregularity information. The proficiency of secondary measures such as TotalEntropy and AvgEntropy to classify physiological data has been demonstrated in this work. Any such secondary statistics of analysis can be obtained from a given entropy profile to suit the purpose of application.
  In general, SampEn estimation is bound by data length limitations. The procedure cannot accurately handle signals of length below 1000 samples. As a consequence, multiscale SampEn analysis, that further constricts data length as part of its formulation, remains inapplicable on short-length physiological signals. When augmented with entropy profiling, we see that the process of multiscaling becomes equipped to handle even the short-length signals.
3. Computational efficiency:
  Traditional SampEn, calculated at a single *r* value, takes an execution time of $o(N^{2})$ [69]. As an attempt to compare this with the execution time of the cumulative histogram method of SampEn profiling, we design the following experiment. Consider the age-based HRV data set used in this study. HRV signal of length 300 from each of the 40 subjects (20 elderly and 20 young) is taken. On each signal, first the CHM is applied. Here, CHM will provide us (a) the data specific range and resolution of *r* and (b) the complete SampEn profile of the signal. The time taken for CHM execution/profile generation per signal is noted down and tabulated in columns 4 and 7 of Table 3. Additionally, from the *r* range and resolution obtained, we get to know the number of unique *r* values to be used for profile generation. This number is denoted as *n* and is tabulated in columns 2 and 5 of Table 3.
  Now, using *n* estimated from the above procedure, we try and generate SampEn profiles following the traditional approach. In other words, SampEn estimation will be repeated *n* times, each time with a unique *r*. The time taken for this execution is tabulated in columns 3 and 6 of Table 3.
  It can be seen that the execution times involving CHM are much less compared to those involving the traditional approach. This is true for both the elderly and young populations of HRV data. For the elderly population, the mean±SD of execution times are 1.57±1.08 s and 0.09±0.02 s respectively for traditional approach and CHM. In the young population—2.66±1.16 s and 0.11±0.02 s. The traditional way of generating a complete SampEn profile is far more computationally expensive than the CHM.
  To give a more comprehensible explanation, say we need to compute SampEn at “*n*” number of *r* values, in order to produce a “complete” profile. The traditional approach will do this by repeating a run of SampEn estimation *n* number of times, each time using a unique *r*. This implies the total execution time will be $o(N^{2}n)$. Now, the higher the value of *n*, the greater the execution time. CHM on the other hand, will do this by simultaneously generating SampEn corresponding to all *r* values in a single run of the algorithm (as explained in Section 5.3). This is irrespective of the value of *n* (small or large). The total execution time will therefore remain $o(N^{2})$.
  CHM involves matrix operations and the concept of cumulative distribution to generate an entropy profile. This makes the approach computationally more efficient than the traditional approaches. We see that traditionally, looped operations have been used to compute entropy at multiple *r* values, increasing computational load.
  It is to be noted that the computational benefit demonstrated above may vary with the computing platform used. Our results have been validated in MATLAB where the kernel loops are optimized compared to traditional loops.

## 8. Conclusions

In KS-entropy methods, the ambiguity of *r*-selection has remained an unresolved issue for over two decades now. The impact of the problem has greatly been felt when dealing with short-length recordings of HRV signals to extract irregularity or complexity based information. By removing the need for a manual *r*-selection process in entropy methods, this work has opened up new avenues in short-length HRV analysis. The proposed novel approach of entropy profiling being accurate, reliable and computationally efficient, demonstrates great potential for high-quality information retrieval from HRV signals as short as one minute recordings. Additionally, the contribution of entropy profiling to short-length HRV analysis indicates 5y3 possibility to incorporate real-time HRV irregularity/complexity measurement in modern wearable devices, e.g., a wrist watch or mobile phone with a built-in HRV analysis module. This will be of significant research potential in the domain of smart health monitoring.

Future Research Directions
  1. Data driven selection of embedding dimension *m*: Similarly to the case of *r*, embedding dimension *m* is yet another parameter that limits entropy methods in the analysis of short-term data. Though not as critical as *r*, *m*-selection also requires a certain extent of discretion when used in entropy estimations. A perfectly appropriate *m* value can greatly help improve information retrieval from short-length HRV signals. Since the right choice of *m* is as ambiguous as that of *r*, we will need methods to help us choose the appropriate *m*. Thinking along the same lines as dealing with *r*, it is best if we are able to completely eliminate the need to choose *m* from entropy estimations. The prospect of eliminating embedding dimension (*m*) from entropy formulations is the primary future target. Following an elaborate survey on the selection, use and impacts of varying embedding dimension (*m*) on entropy, a geometrical explanation of the parameter’s properties and behavior is obtainable. This helps formulate a relation between data and their potential range of *m* values. Then the demand is for a data-driven algorithm capable of generating these potential *m* values automatically. In this way, once the complete entropy profile is obtained with respect to *m*, beneficial measures of regularity or complexity can be extracted from it. The physiological relevance of *m*-selection in the context of HRV analysis can also be another stream of research here.
  2. Extending the method for any given physiological signal: A significant extension of our work will be meant to make our methods compatible for use with any kind of physiological signal, not just HRV. Now, an important aspect to be considered here is the frequency of the input signals. For instance, some physiological signals, such as the PPG (photoplethysmographic) signals, are low-frequency in nature, while ones such as EEG (electroencephalographic) signals are of more high frequency components. It is essential to study how our entropy profiling method responds to changes in input signal frequencies. Since the method is entirely dependent on signal dynamics, a frequency-based study makes an indispensable future goal.
  3. Cross entropy analysis on multi-variate signals: Looking for correlations in bi-variate or multi-variate physiological time series is a research domain that has picked up speed in recent years. Finding synchrony between two different physiological signals (coming from the same system) may reveal significant information about the underlying physiological system. For this purpose, it has been a common practice to employ cross-entropy versions of approximate and sample entropy. Implementing cross-entropy profiling instead of estimations could prove beneficial here too. Using CHM and thereby entropy profiling to perform multi-variate signal analysis will improve prospects of analysis on short-length multivariate signals. This could be an important direction of future experiments and validation attempts.
  4. Simulation trials using different models: HRV is a non-stationary process, with long-range and short-range dependencies. Entropy profiling assumes stationarity in the signal, when evaluating short segments of the data. However, if it is to be used on longer lengths of data, signal attributes and assumptions may have to change. Signals may become more complex. To test the robustness of the method, it will be significant to run simulations on complex auto-regressive models such as ARFIMA (autoregressive fractionally integrated moving average) [70] and GARCH (generalized autoregressive conditional heteroskedasticity) [27].

MATLAB codes generated as part of our study are available at https://github.com/radhagayathri/Entropy-Codes. These can be used with proper citations included.

## Figures and Tables

**Table 1:** Classification of healthy “elderly” and healthy “young” HRV data at different data lengths *N* [49].

*N*	$H_{S}$		$H_{F}$		$H_{\mathrm{TS}}$		$H_{\mathrm{AS}}$	
	*p* Value	AUC	*p* Value	AUC	*p* Value	AUC	*p* Value	AUC
50	NA	NA	NS	0.62	0.00051	0.82	0.0012	0.80
100	NA	NA	NS	0.68	0.00022	0.84	0.0106	0.74
150	NS	0.54	NS	0.64	0.00022	0.84	0.0179	0.72
200	NS	0.62	0.0315	0.70	0.00004	0.88	0.0077	0.75
250	NS	0.65	0.0193	0.72	0.00004	0.88	0.0084	0.75
300	NS	0.68	0.0098	0.74	0.00005	0.88	0.0207	0.72

**Table 2:** Classification of “healthy” and “arrhythmic” HRV data at different data lengths *N* [49].

*N*	$H_{S}$		$H_{F}$		$H_{\mathrm{TS}}$		$H_{\mathrm{AS}}$	
	*p* Value	AUC	*p* Value	AUC	*p* Value	AUC	*p* Value	AUC
50	NA	NA	NS	0.61	1.88 × 10^−8^	0.95	0.0001	0.81
100	NA	NA	NS	0.65	1.73 × 10^−8^	0.95	0.0005	0.78
150	NS	0.60	0.0098	0.71	8.82 × 10^−9^	0.96	0.0007	0.77
200	NS	0.61	0.0016	0.75	1.34 × 10^−8^	0.96	0.0002	0.80
250	NS	0.65	0.0022	0.75	2.85 × 10^−8^	0.95	0.0034	0.74
300	NS	0.66	0.0021	0.75	1.46 × 10^−8^	0.96	0.0058	0.72

**Table 3:** Execution times of (1) the traditional approach and (2) CHM, to generate a complete SampEn profile; HRV signals, N=300, from elderly and young populations of subjects are used. *n* represents the number of unique *r* values [49].

Healthy “Elderly”				Healthy “Young”		
Signal	n	Execution Time (s)		n	Execution Time (s)	
		Traditional Approach	CHM		Traditional Approach	CHM
1	127	1.08	0.08	280	2.53	0.10
2	143	1.17	0.09	352	3.14	0.11
3	127	1.10	0.08	137	1.24	0.09
4	323	2.75	0.12	340	3.01	0.12
5	31	0.29	0.07	275	2.41	0.10
6	78	0.74	0.08	314	2.76	0.12
7	91	0.77	0.09	508	4.44	0.16
8	119	1.15	0.08	209	1.91	0.08
9	356	3.23	0.12	135	1.11	0.08
10	144	1.23	0.08	197	1.72	0.09
11	460	4.10	0.14	201	1.84	0.09
12	265	2.36	0.10	384	3.37	0.13
13	62	0.63	0.07	123	1.08	0.09
14	133	1.18	0.09	96	1.12	0.08
15	64	0.61	0.08	407	3.53	0.12
16	186	1.65	0.09	251	2.32	0.11
17	99	0.87	0.08	321	2.89	0.12
18	267	2.24	0.10	415	3.71	0.12
19	396	3.49	0.12	559	5.26	0.16
20	97	0.78	0.08	424	3.75	0.12

## References

[1] Estela K.B., Mark R., Paul F., Joseph R. (1995). Heart Rate Variability in Health and Disease. Scand. J. Work Environ. Health.

[2] Huikuri H.V., Makikallio T., Airaksinen K., Mitrani R., Castellanos A., Myerburg R.J. (1999). Measurement of heart rate variability: A clinical tool or a research toy?. J. Am. Coll. Cardiol..

[3] Sandercock G.R., Bromley P.D., Brodie D.A. (2005). Review: The reliability of short-term measurements of heart rate variability. Int. J. Cardiol..

[4] Malik M., Bigger J.T., Camm A.J., Kleiger R.E., Malliani A., Moss A.J., Schwartz P.J. (1996). Heart rate variability: Standards of measurement, physiological interpretation and clinical use. Task Force of the European Society of Cardiology and the North American Society of Pacing and Electrophysiology. Circulation.

[5] Goldberger A.L., West B.J. (1987). Applications of nonlinear dynamics to clinical cardiology. Ann. N. Y. Acad. Sci..

[6] Voss A., Schulz S., Schroeder R., Baumert M., Caminal P. (2009). Methods Derived from Nonlinear Dynamics for Analysing Heart Rate Variability. Philos. Trans. Math. Phys. Eng. Sci..

[7] Voss A., Schroeder R., Heitmann A., Peters A., Perz S. (2015). Short Term Heart Rate Variability Influence of Gender and Age in Healthy Subjects. PLoS ONE.

[8] Chen W., Zhuang J., Yu W., Wang Z. (2009). Measuring complexity using FuzzyEn, ApEn, and SampEn. Med. Eng. Phys..

[9] Lu S., Chen X., Kanters J.K., Solomon I.C., Chon K.H. (2008). Automatic Selection of the Threshold Value for Approximate Entropy. IEEE Trans. Biomed. Eng..

[10] Li P., Liu C., Li K., Zheng D., Liu C., Hou Y. (2015). Assessing the complexity of short-term heartbeat interval series by distribution entropy. Med. Biol. Eng. Comput..

[11] Grassberger P., Procaccia I. (1983). Measuring the strangeness of strange attractors. Physica D.

[12] Eckmann J.P., Ruelle D. (1985). Ergodic theory of chaos and strange attractors. Rev. Mod. Phys..

[13] Pincus S.M. (1991). Approximate entropy as a measure of system complexity. Proc. Natl. Acad. Sci. USA.

[14] Pincus S.M., Gladstone I.M., Ehrenkranz R.A. (1991). A regularity statistic for medical data analysis. J. Clin. Monit..

[15] Al-Angari H.M., Sahakian A.V. (2007). Use of sample entropy approach to study heart rate variability in obstructive sleep apnea syndrome. IEEE Trans. Biomed. Eng..

[16] Richman J., Moorman J. (2000). Physiological time-series analysis using approximate entropy and sample entropy. Am. J. Physiol..

[17] Xie H.B., He W.X., Liu H. (2008). Measuring time series regularity using nonlinear similarity-based sample entropy. Phys. Lett. A.

[18] Xie H.B., Chen W.T., He W.X., Liu H. (2011). Complexity analysis of the biomedical signal using fuzzy entropy measurement. Appl. Soft Comput. J..

[19] Costa M., Goldberger A.L., Peng C.K. (2002). Multiscale entropy analysis of complex physiologic time series. Phys. Rev. Lett..

[20] Udhayakumar R.K., Karmakar C., Palaniswami M. (2019). Multiscale Entropy Profiling to Estimate Complexity of Heart Rate Dynamics. Phys. Rev. E.

[21] Udhayakumar R., Karmakar C., Palaniswami M. Cross Entropy Profiling to Test Pattern Synchrony in Short-Term Signals. Proceedings of the 2019 41st Annual International Conference of the IEEE Engineering in Medicine and Biology Society (EMBC).

[22] Amigó J.M. (2012). The equality of Kolmogorov–Sinai entropy and metric permutation entropy generalized. Phys. D Nonlinear Phenom..

[23] Pincus S.M. (2000). Irregularity and asynchrony in biologic network signals. Methods Enzymol..

[24] Liu C., Liu C., Shao P., Li L., Sun X., Wang X., Liu F. (2011). Comparison of different threshold values *r* for approximate entropy: Application to investigate the heart rate variability between heart failure and healthy control groups. Physiol. Meas..

[25] Mayer C.C., Bachler M., Hörtenhuber M., Stocker C., Holzinger A., Wassertheurer S. (2014). Selection of entropy-measure parameters for knowledge discovery in heart rate variability data. BMC Bioinform..

[26] Yentes J.M., Hunt N., Schmid K.K., Kaipust J.P., McGrath D., Stergiou N. (2013). The appropriate use of approximate entropy and sample entropy with short data sets. Ann. Biomed. Eng..

[27] Liu L.Z., Qian X.Y., Lu H.Y. (2010). Cross-sample entropy of foreign exchange time series. Phys. A Stat. Mech. Its Appl..

[28] Castiglioni P., Di Rienzo M. How the threshold *r* influences approximate entropy analysis of heart-rate variability. Proceedings of the 2008 Computers in Cardiology.

[29] Castiglioni P., Zurek S., Piskorski J., Kosmider M., Guzik P., Ce E., Rampichini S., Merati G. Assessing Sample Entropy of physiological signals by the norm component matrix algorithm: Application on muscular signals during isometric contraction. Proceedings of the 2013 35th Annual International Conference of the IEEE Engineering in Medicine and Biology Society (EMBC).

[30] Boskovic A., Loncar-Turukalo T., Japundzic-Zigon N., Bajic D. The flip-flop effect in entropy estimation. Proceedings of the 2011 IEEE 9th International Symposium on Intelligent Systems & Informatics (SISY).

[31] Lake D.E., Moorman J.R. (2011). Accurate estimation of entropy in very short physiological time series: The problem of atrial fibrillation detection in implanted ventricular devices. Am. J. Physiol..

[32] Liu C., Oster J., Reinertsen E., Li Q., Zhao L., Nemati S., Clifford G.D. (2018). A comparison of entropy approaches for AF discrimination. Physiol. Meas..

[33] Cysarz D., Lange S., Matthiessen P.F., van Leeuwen P. (2007). Regular heartbeat dynamics are associated with cardiac health. Am. J. Physiol. Regul. Integr. Comp. Physiol..

[34] Kumar M., Pachori R.B., Acharya U.R. (2017). Use of Accumulated Entropies for Automated Detection of Congestive Heart Failure in Flexible Analytic Wavelet Transform Framework Based on Short-Term HRV Signals. Entropy.

[35] Fleisher L.A., Pincus S.M., Rosenbaum S.H. (1993). Approximate entropy of heart rate as a correlate of postoperative ventricular dysfunction. Anesthesiology.

[36] Signorini M.G. Nonlinear Analysis of Heart Rate Variability Signal: Physiological Knowledge and Diagnostic Indications. Proceedings of the Annual International Conference—IEEE Engineering in Medicine and Biology Society.

[37] Liu G., Wang Q., Chen S., Zhou G., Chen W., Wu Y. (2014). Robustness evaluation of heart rate variability measures for age gender related autonomic changes in healthy volunteers. Australas. Phys. Eng. Sci. Med..

[38] Voss A., Heitmann A., Schroeder R., Peters A., Perz S. (2012). Short-term heart rate variability–age dependence in healthy subjects. Physiol. Meas..

[39] Voss A., Schroeder R., Fischer C., Heitmann A., Peters A., Perz S. Influence of age and gender on complexity measures for short term heart rate variability analysis in healthy subjects. Proceedings of the 2013 35th Annual International Conference of the IEEE Engineering in Medicine and Biology Society (EMBC).

[40] Lake D.E., Richman J.S., Griffin M.P., Moorman J.R., Mosconi G., Carnevali O., Habibi H. (2002). Sample entropy analysis of neonatal heart rate variability. Am. J. Physiol..

[41] Xinnian C., Solomon I., Chon K. Comparison of the Use of Approximate Entropy and Sample Entropy: Applications to Neural Respiratory Signal. Proceedings of the 2005 IEEE Engineering in Medicine and Biology 27th Annual Conference.

[42] Costa M., Peng C.K., Goldberger A.L., Hausdorff J.M. (2003). Multiscale entropy analysis of human gait dynamics. Physica A.

[43] Costa M., Goldberger A.L., Peng C.K. (2005). Multiscale entropy analysis of biological signals. Phys. Rev. E Stat. Nonlin. Soft Matter Phys..

[44] Acharya U.R., Joseph K.P., Kannathal N., Lim C.M., Suri J.S. (2006). Heart rate variability: A review. Med. Biol. Eng. Comput..

[45] Thuraisingham R.A., Gottwald G.A. (2006). On multiscale entropy analysis for physiological data. Physica A.

[46] Liu C., Li K., Zhao L., Liu F., Zheng D., Liu C., Liu S. (2013). Analysis of heart rate variability using fuzzy measure entropy. Comput. Biol. Med..

[47] Ji L., Li P., Li K., Wang X., Liu C. (2015). Analysis of short-term heart rate and diastolic period variability using a refined fuzzy entropy method. Biomed. Eng. Online.

[48] Liu Y., Lin Y., Wang J., Shang P. (2018). Refined generalized multiscale entropy analysis for physiological signals. Physica A.

[49] Udhayakumar R., Karmakar C., Palaniswami M. (2018). Understanding Irregularity Characteristics of Short-term HRV Signals using Sample Entropy Profile. IEEE Trans. Biomed. Eng..

[50] Udhayakumar R.K., Karmakar C., Palaniswami M. (2016). Approximate entropy profile: A novel approach to comprehend irregularity of short-term HRV signal. Nonlinear Dyn..

[51] Pincus S.M., Goldberger A.L. (1994). Physiological time-series analysis: What does regularity quantify?. Am. J. Physiol..

[52] Pincus S.M., Viscarello R.R. (1992). Approximate entropy: A regularity measure for fetal heart rate analysis. Obstet. Addit. Gynecol..

[53] Pincus S. (1995). Approximate entropy (ApEn) as a complexity measure. Chaos.

[54] Ryan S.M., Goldberger A.L., Pincus S.M., Mietus J., Lipsitz L.A. (1994). Gender- and age-related differences in heart rate dynamics: Are women more complex than men?. J. Am. Coll. Cardiol..

[55] Porta A., Gnecchi-Ruscone T., Tobaldini E., Guzzetti S., Furlan R., Montano N. (2007). Progressive decrease of heart period variability entropy-based complexity during graded head-up tilt. J. Appl. Physiol..

[56] Madalena D.C., Chung-Kang P., Ary L.G. (2008). Multiscale Analysis of Heart Rate Dynamics: Entropy and Time Irreversibility Measures. Cardiovasc. Eng. Int. J..

[57] Ahmed M.U., Mandic D.P. (2011). Multivariate multiscale entropy: A tool for complexity analysis of multichannel data. Phys. Rev. E Stat. Nonlin. Soft Matter Phys..

[58] Costa M., Goldberger A.L., Peng C.K. (2002). Multiscale entropy to distinguish physiologic and synthetic RR time series. Comput. Cardiol..

[59] Goldberger A.L. (1991). Is the normal heartbeat chaotic or homeostatic?. Physiology.

[60] Chang Y.C., Tsao J.H., Wu H.T., Chen H.R., Liu A.B., Yeh J.J., Lo M.T., Tang C.J., Tsai I.T., Sun C.K. (2014). Application of a Modified Entropy Computational Method in Assessing the Complexity of Pulse Wave Velocity Signals in Healthy and Diabetic Subjects. Entropy.

[61] Wu S.D., Wu C.W., Lee K.Y., Lin S.G. (2013). Modified multiscale entropy for short-term time series analysis. Physica A.

[62] Udhayakumar R., Karmakar C., Li P., Wang X., Palaniswami M. (2020). Modified Distribution Entropy as a Complexity Measure of Heart Rate Variability (HRV) Signal. Entropy.

[63] Freedman D., Diaconis P. (1981). On the histogram as a density estimator:L2 theory. Z. Wahrscheinlichkeitstheorie Verwandte Geb..

[64] Udhayakumar R., Karmakar C., Palaniswami M. Secondary measures of regularity from an entropy profile in detecting Arrhythmia. Proceedings of the 2017 39th Annual International Conference of the IEEE Engineering in Medicine and Biology Society (EMBC).

[65] Udhayakumar R., Karmakar C., Palaniswami M. Entropy Profiling to Detect ST Change in Heart Rate Variability Signals. Proceedings of the 2019 41st Annual International Conference of the IEEE Engineering in Medicine and Biology Society (EMBC).

[66] Goldberger A.L., Amaral L.A.N., Glass L., Hausdorff J.M., Ivanov P.C., Mark R.G., Mietus J.E., Moody G.B., Peng C.K., Stanley H.E. (2000). PhysioBank, PhysioToolkit, and PhysioNet: Components of a New Research Resource for Complex Physiologic Signals. Circulation.

[67] Iyengar N., Peng C.K., Morin R., Goldberger A.L., Lipsitz L.A. (1996). Age-related alterations in the fractal scaling of cardiac interbeat interval dynamics. Am. J. Physiol..

[68] Moody G.B., Mark R.G. (2001). The impact of the MIT-BIH arrhythmia database. IEEE Eng. Med. Biol. Mag..

[69] Pan Y.H., Wang Y.H., Liang S.F., Lee K.T. (2011). Fast computation of sample entropy and approximate entropy in biomedicine. Comput. Methods Programs Biomed..

[70] Contreras-Reyes J., Palma W. (2013). Statistical Analysis of Autoregressive Fractionally Integrated Moving Average Models in R. Comput. Stat..

